# Risk Factors for TB/HIV Coinfection and Consequences for Patient Outcomes: Evidence from 241 Clinics in the Democratic Republic of Congo

**DOI:** 10.3390/ijerph18105165

**Published:** 2021-05-13

**Authors:** Gulzar Hussain Shah, Raimi Ewetola, Gina Etheredge, Lievain Maluantesa, Kristie Waterfield, Elodie Engetele, Apolinaire Kilundu

**Affiliations:** 1 Jiann-Ping Hsu College of Public Health, Georgia Southern University, Statesboro/Savannah, GA 30460, USA; kwaterfield@georgiasouthern.edu; 2 Division of Global HIV and Tuberculosis, CDC, Atlanta, GA 30333, USA; hcx6@cdc.gov; 3 FHI 360, Washington, DC 20009, USA; GEtheredge@fhi360.org; 4 FHI 360, Kinshasa 1015, Democratic Republic of the Congo; LMaluantesa@fhi360.org (L.M.); EEngetele@fhi360.org (E.E.); 5 National AIDS Control Program (PNLS), Democratic Republic of the Congo; apogkilundu@gmail.com

**Keywords:** TB/HIV coinfection, Democratic Republic of Congo, HIV loss to follow-up, viral load suppression, death of HIV/AIDS patient

## Abstract

(1) Background: In resource-limited countries, patients with tuberculosis (TB)/HIV coinfection commonly face economic, sociocultural, and behavioral barriers to effective treatment. These barriers manifest from low treatment literacy, poverty, gender inequality, malnutrition, societal stigmas regarding HIV, and an absence of available care. It is critical for intervention programs to understand and assist in overcoming these barriers and any additional risks encountered by patients with TB/HIV coinfection. This study analyzes variation in TB/HIV coinfection and risks of negative outcomes among patients with TB/HIV coinfection compared to those without coinfection. (2) Methods: This quantitative study used data from 49,460 patients receiving ART from 241 HIV/AIDS clinics in Haut-Katanga and Kinshasa, two provinces in the Democratic Republic of Congo. Chi-square and logistic regression analysis were performed. (3) Results: Significantly higher proportions of patients with TB/HIV coinfection were men (4.5%; women, 3.3%), were new patients (3.7%; transferred-in, 1.6%), resided in the Kinshasa province (4.0%; Haut-Katanga, 2.7%), and were in an urban health zone (3.9%) or semi-rural health zone (3.1%; rural, 1.2%). Logistic regression analysis showed that after controlling for demographic and clinical variables, TB/HIV coinfection increased the risk of death (adjusted odds ratio (AOR), 2.26 (95% confidence interval (CI): 1.94–2.64)) and LTFU (AOR, 2.06 (95% CI: 1.82–2.34)). TB/HIV coinfection decreased the odds of viral load suppression (AOR, 0.58 (95% CI: 0.46–0.74)). (4) Conclusions: TB/HIV coinfection raises the risk of negative outcomes such as death, LTFU, and lack of viral load suppression. Our findings can help HIV clinics in Democratic Republic of Congo and other African countries to customize their interventions to improve HIV care and reduce care disparities among patients.

## 1. Introduction

In sub-Saharan Africa, tuberculosis (TB) and HIV coinfection has been associated with high morbidity and mortality rates among people living with HIV/AIDS [1,2]. According to the World Health Organization (WHO), Africa Region has the highest proportion of TB/HIV coinfection and of the 251,000 deaths from HIV-associated TB in 2019, approximately 84% were in sub-Saharan Africa [3]. The estimated epidemiological burden in Central and Western Africa finds six of these countries on the list of the 30 highest TB burdened countries; for 2019, TB/HIV co-infection cases in these six countries range from 5800 (Republic of the Congo) to 46,000 (Nigeria), and HIV-positive TB mortalities ranged from 2200 (Republic of the Congo) to 27,000 (Nigeria) [3]. Democratic Republic of Congo (DRC) is one of 14 countries that appear on all three of the WHO’s High-Burden Country lists for TB, TB/HIV, and multidrug-resistant TB [3]. The 2019 data from DRC also showed that 9.1% of the 74,450 newly enrolled people in HIV treatment were also diagnosed with TB during the same year [3]. Because DRC has limited health care resources, people living with HIV/AIDS (PLWH) have limited access to care and suboptimal quality of care. HIV programs and health centers are subject to medication stock-outs, social inequity, staffing shortages, and lack of information-gathering tools [4,5,6,7]. The patient monitoring efforts also face challenges such as poor collection of samples with late results and sometimes missing viral load results, inadequate biological monitoring, low availability of normative documents, low motivation of providers, and limited access to patients’ current and ongoing viral load data [4,5,6,7]. These challenges coupled with patient characteristics, such as coinfections, WHO clinical stage, and viral load suppression status, contribute to loss to follow-up (LTFU), patients transferring out of care, and death [4,5,6,7,8,9]. According to WHO, of the approximately 38 million PLWH globally, 25.7 million live in sub-Saharan Africa, and approximately 400,000 PLWH who live in the West and Central regions of sub-Saharan Africa reside in DRC [10].

Since 2002, strategies to control the HIV epidemic have been proactively devised and implemented by the DRC Ministry of Health. The partnerships between the DRC Ministry of Health, the Centers for Disease Control and Prevention (CDC), and non-governmental organizations have significantly decreased HIV mortality rates in patients of all ages in DRC since 2000 (41,000 HIV-related deaths compared to 16,339 HIV-related deaths in 2018) [10,11,12,13]. However, despite this progress, the mortality rate remains high due to coinfections such as TB/HIV [7,14,15]. This is not surprising because TB is the fourth leading cause of death and the fifth leading cause of premature death (death that occurs before the average age of death) among all persons living in DRC [14]. In high-burden countries, such as DRC, the number of undetected TB cases can exceed 50% [3]. In 2016, the estimated TB incidence rate in DRC was 323 cases per year per 100,000 inhabitants in a population of 85 million, with approximately 125,000 new cases of TB that went undetected that year [11].

WHO’s End TB Strategy recommends a 95% decrease in TB deaths and a 90% decrease in TB incidence by 2035, compared with levels in 2015 [3]. The integration between a country’s TB and HIV care services affects program monitoring, treatment evaluation, and referral from one program to another [16]. In many high-burden countries, such as DRC, Kenya, Mozambique, and South Africa, integration of TB and HIV services ensure that TB patients are tested for HIV and HIV patients are screened for TB. Such integration has decreased the number of deaths each year, increased TB cure rates, improved case detection, and increased identification and treatment of drug-resistant TB [16]. The DRC Ministry of Health, with support from United States CDC, has comprehensively integrated HIV and TB treatment in the Haut-Katanga and Kinshasa provinces. Currently, of the approximately 20,000 incident TB-HIV cases in DRC, only 10% of notified patients with TB are aware of their HIV-positive status; however, 82% of TB patients who are aware of their HIV-positive status are receiving antiretroviral therapy (ART) [3].

The HIV and TB programs and health centers face several challenges that hinder DRC Ministry of Health efforts to strengthen the public health systems and control both the HIV and TB epidemics [11]. Understanding the sociological factors that affect TB/HIV coinfection rates at outpatient clinics in the DRC will help inform the decision-making processes regarding prevention, treatment, service delivery, and resource allocation. We examined patient characteristics associated with TB/HIV coinfection and patient outcomes at outpatient clinics in Haut-Katanga and Kinshasa. We investigated whether the proportion of patients with TB/HIV coinfection vary by the demographic and clinical characteristics, such as age, sex, province, urban/rural location of the health zone, ART initiation mode, and ART duration, and whether TB/HIV coinfection influences patient outcomes, such as death, LTFU, and viral load suppression.

## 2. Materials and Methods

### 2.1. Data

For this research, we used a quantitative, retrospective, observational study design. We obtained data from 241 HIV/AIDS clinics in 23 health zones in Haut-Katanga (n = 11) and Kinshasa (n = 12). The dataset for this study comprised 49,460 persons living with HIV who were receiving ART from 1 January 2014, to 31 May 2019. Tier.net data were collected daily at the health facility level. Each month the health facility sends data to the health zone that compiles data from all the health facilities. Monthly, the health zones send data to the province, which in turn shared those data to the national level repository, using the electronic system. This dataset with a limited number of demographic and clinical variables was obtained from the National HIV/AIDS Program (PNLS) national electronic patient management system, which manages the HIV clinics owned by the DRC government supported by CDC implementing partners (ICAP and EGPAF) via the President’s Emergency Plan for AIDS Relief (PEPFAR). Georgia Southern University’s Institutional Review Board approved the study under the project protocol number H l9260.

### 2.2. Measures

The primary variable of interest was TB/HIV coinfection, which was first treated as a dependent variable to explore the factors associated with variation in this coinfection and later as a primary independent variable to examine the impact of coinfection on patient outcomes. At the HIV clinics included in the study, the TB diagnosis is performed by microscopy, X-ray, Xpert. The GenXpert can be described as: (1) Cepheid GeneXpert IV analyzer with starter kit laptop. Tabletop: 24 kg with laptop. Technology: Nested real-time reverse transcriptase PCR, reference GXIV-4-D. (2) GeneXpert IV with desktop, Tabletop: 24 kg with laptop. The GenXpert technology is nested real-time reverse transcriptase PCR, reference GXIV-4-L. Culture is done for multiresistant cases. The HIV infection diagnosis is performed by rapid test according to the national algorithm. The variable TB status was originally coded as 0 (unknown), 1 (not screened), 2 (on TB treatment at another facility), 3 (on TB treatment at this facility), 4 (no symptoms), 5 (symptoms with no sputum (mucus)), 6 (symptoms with sputum (mucus)), and 7 (not detected). For our analysis, we re-coded the variable TB coinfection as a dichotomous variable, with original category-codes 2, 3, 5, and 6 as 1 indicating “TB present”; original category-codes 4 and 7 were coded as 0 to indicate “TB not present”; and records with original codes 0 (unknown) and 1 (not screened) were excluded from our analysis.

Three outcomes were computed from the original variable “patient outcome” coded as 0 (died), 1 (LTFU), 2 (transferred out), and 3 (presently in treatment). Death as an outcome was operationalized by recoding the original category 0 as 1 to indicate “died,” and the other categories (LTFU, still in care, and transferred out) were recoded as 0. The outcome LTFU was computed by excluding category 0 from the analysis, keeping the original category-code 1 to indicate LTFU, and others as 0 to note “in care or transferred out.” Viral load suppression was recoded as a dichotomous variable with the categories <1000 copies/mL and ≥1000 copies/mL, per WHO recommendations [17].

Independent variables for the bivariate association with TB coinfection included two demographic variables: patient sex and age at the time of the first visit. The variable age of patient was computed using date of first visit and patient’s date of birth. This variable was dichotomized as aged <15 years and ≥15 years because the two groups have distinct challenges. We also included three independent variables pertaining to the geographic location of the clinic: province (Haut-Katanga or Kinshasa), health zone (n = 23), and rural/urban status of the health zone (rural, semi-rural, or urban). We also included ART initiation mode (new patient and transferred in) and ART duration (months) at the time of the last reported visit, which was computed subtracting ART start date from the date of the last visit for ART. ART duration was recoded into quartiles (<3.23 months, 3.23–14.52 months, 14.53–40.37 months, and >40.37 months).

### 2.3. Analytical Methods

We used descriptive statistics, such as frequencies and percentages, for all independent and dependent variables. To assess the bivariate associations of categorical independent variables with TB/HIV coinfection as the dichotomous dependent variable, we performed chi-square tests. Three separate logistic regression analyses were performed to estimate the impact of TB status on three dichotomous dependent variables: death (vs. in care, transferred out, or LTFU), LTFU (vs. in care or transferred out), and viral load suppression, after controlling for other variables such as patient’s sex, age, ART initiation mode, ART duration, province, health zone, and rural/urban status of health zone. The missing data were handled using the listwise deletion approach. All analyses for this study were performed using SPSS, version 25.0 (IBM Corporation, Armonk, NY, USA) [18]. *p*-values < 0.05 were considered to indicate statistically significant associations.

## 3. Results

Descriptive statistics for demographic and clinical characteristics are presented in Table 1. Our data showed that 5.8% (i.e., 2851 of 49,460) of patients receiving ART at HIV/AIDS clinics died, 16.6% (7737 of 46,609) were LTFU, and 81.2% (12,281 of 15,133) had viral load suppression (Table 1). Coinfection of TB/HIV was present in 3.6% of the patients. Our study population included 69.0% females and 31.0% males. One in 10 (10.3%) were younger than 15 years of age. Of the patients initiating ART, 95.2% were new and 4.8% transferred in from other facilities. Having divided the distribution into quartiles, one-fourth of our study population had been on ART for <3.23 months, and the same proportions for 3.23 to 14.52 months, 14.53 to 40.37 months, and >40.37 months. Rural zones accounted for 5.6%, semi-rural for 11.7%, and urban zones for 82.7% of the study population.

### 3.1. Correlates of TB/HIV Coinfection

Bivariate associations of patient clinical and demographic characteristics are presented in Table 2. TB/HIV coinfection was significantly higher in men (4.5%) than women (3.3%) and in new patients (3.7%) than in patients who transferred to the clinic (1.6%). TB/HIV coinfection was significantly lower in patients in Haut-Katanga (2.7%) than in Kinshasa (4.0%) and in rural (1.2%) and semi-rural (3.1%) health zones than in urban (3.9%) health zones. We found a significant but non-linear association between ART duration and TB/HIV coinfection; the longer the duration of ART, the lower the odds of developing TB coinfection.

### 3.2. TB/HIV Coinfection and Factors Aaffecting Risk of Death

After controlling for other variables in the model (Table 3), odds of death were significantly higher for patients with TB/HIV coinfection (adjusted odds ratio (AOR), 2.26 (95% confidence interval (CI): 1.94–2.64)). Odds of death were significantly lower for women than men (AOR, 0.36 (95% CI: 0.34–0.39)). New patients had significantly lower odds of death than patients who transferred into the clinic (AOR, 0.08 (95% CI: 0.07–0.09)). Odds of death were significantly lower for patients who had received ART for 3.23–14.52 months (AOR, 0.81 (95% CI: 0.71–0.93)) and for 14.53–40.37 months (AOR, 0.59 (95% CI: 0.52–0.67)) than those who had received ART for >40.37 months; however, patients who had received ART for <3.23 months (AOR, 2.32 (95% CI: 2.08–2.59)) had significantly higher odds of death than those who had received ART for >40.37 months. Odds of death were significantly higher for patients in the Haut-Katanga province versus Kinshasa (AOR, 1.14 (95% CI: 1.04–1.25)), and for patients in semi-rural health zones compared to those in urban (AOR, 1.20 (95% CI: 1.07–1.35)). The risk of death was significantly lower for patients in rural health zones compared to those in urban areas (AOR, 0.24 (95% CI: 0.17–0.33)). Age of the patient had no significant association with the risk of death.

### 3.3. TB/HIV Coinfection and Factors Affecting Odds of LTFU

Risk of LTFU was significantly higher for patients with TB/HIV coinfection (AOR, 2.06 (95% CI: 1.82–2.34)). Odds of LTFU were significantly lower for women than men (AOR, 0.72 (95% CI: 0.68–0.76)). Patients younger than 15 years also had higher odds of LTFU (AOR, 1.25 (95% CI: 1.14–1.37)). New patients had significantly lower odds of LTFU when compared to patients that transferred into the clinic (AOR, 0.15 (95% CI: 0.14–0.16)). Odds of LTFU were significantly higher for patients who had received ART for <3.23 months (AOR, 2.83 (95% CI: 2.61–3.08)) and 3.23–14.52 months (AOR, 1.35 (95% CI: 1.24–1.48)) than those who received ART for >40.37 months. Odds of LTFU were significantly lower for patients in Haut-Katanga compared to those in Kinshasa (AOR, 0.50 (95% CI: 0.46–0.54)) and for patients in rural (AOR, 0.04 (95% CI: 0.02–0.08)) and semi-rural (AOR, 0.51 (95% CI: 0.46–0.57)) health zones compared to those in urban areas.

### 3.4. TB/HIV Coinfection and Factors Affecting Odds of Viral Load Suppression

TB/HIV coinfection lowered the odds of viral load suppression (AOR, 0.58 (95% CI: 0.46–0.74)). Odds of viral load suppression were significantly higher for women than men (AOR, 1.82 (95% CI: 1.68–1.96)). Patients younger than 15 years compared to those aged ≥15 years had significantly lower odds of viral load suppression (AOR, 0.44 (95% CI: 0.39–0.50)). New patients had significantly higher odds of viral load suppression than patients who transferred into the clinic (AOR, 2.16 (95% CI: 1.99–2.34)). Odds of viral load suppression were significantly higher for patients who had received ART for 3.23–14.52 months (AOR, 1.35 (95% CI: 1.18–1.55)) or for 14.53–40.37 months (AOR, 1.28 (95% CI: 1.16–1.40)) than those who had received ART for >40.37 months. Odds of viral load suppression were significantly higher for patients in Haut-Katanga than those in Kinshasa (AOR, 2.04 (95% CI: 1.82–2.28)) and for patients in rural health zones compared to those in urban areas (AOR, 1.50 (95% CI: 1.15–1.96)).

## 4. Discussion

We found TB/HIV coinfection in 3.6% of the patients being treated in the 241 HIV/AIDS clinics participating in the study during the study period. Recent cross-sectional studies of TB/HIV coinfection prevalence in sub-Saharan Africa have reported 6.3% among adult patients (aged > 18 years) presenting at TB clinics in Nigeria [1] and 4.3% among adult participants (aged > 15 years) in the national TB prevalence survey administered by the Ministry of Health in Zambia [19]. A retrospective study conducted by Teklu et al. reported a 9% coinfection prevalence rate among all HIV patients in an Advanced Clinical Monitoring of ART cohort in Ethiopia [20]. More females (69.0%) than males (31%) sought care from the 241 HIV/AIDS clinics included in our study, although the male to female ratio in DRC is normal, i.e., 99.7 [21]. While the programs may be specifically targeting more women than men for a variety of reasons, the gender differences in healthcare-seeking behavior due to social and cultural influences may have played a role. Delayed health-seeking behavior among men remains a major challenge in Africa and DRC. As a result, there may be consequences of delay in care-seeking among men with HIV.

We also found significant variations in TB/HIV coinfection by demographic, geographic, and other variables. Such variations indicate that the TB/HIV coinfection services for patients can be improved to avoid healthcare inequities and disparities. To close those gaps and improve services, the HIV programs in Kinshasa and Haut-Katanga provinces could consider adopting a tested and validated quality improvement framework to develop a culture of quality improvement in all aspects of HIV-service provision. Such quality improvement efforts could start with high-risk groups, such as patients with TB/HIV coinfection, to document and address upstream factors associated with TB/HIV coinfection disparities resulting from socioeconomic and other health inequities. Variation in TB/HIV coinfections could be attributed to risk factors such as poor and crowded living conditions, homelessness, drug misuse, alcohol misuse, common mental disorders, and poor diet [1,22,23,24,25].

We found that TB/HIV coinfection increases the risk of negative outcomes for PLWH, including death, LTFU, and lack of viral load suppression. We found that patients with TB/HIV coinfection had a 2.26 times higher risk of death than other PLWH. This increased risk of death may be due to treatment failure, a lack of adherence to anti-TB treatment, or a late diagnosis of TB and/or HIV [26]. Consistent with other studies that have suggested that TB/HIV coinfection increases the risk of LTFU [27,28], we found that persons with TB/HIV coinfection had significantly higher odds of LTFU (AOR, 2.06) than those without TB. Staying in medical care is critical for PLWH to benefit from life-prolonging treatment, which leads to fewer HIV-related complications and improved outcomes [29,30]. Given that lack of treatment, adherence is likely to have social as well as quality-of-care consequences, in addition to quality improvement efforts, HIV/TB programs could consider integrating approaches to reduce TB stigma and improve patients’ compliance [31]. We also found that patients with TB/HIV coinfection were significantly less likely to have viral load suppression (AOR, 0.58).

Our study showed that the risk of death was significantly lower for patients in rural health zones compared to those in urban areas. In general, the in-hospital death rate among TB-HIV co-infected remains high in the DRC, which is around 40%, whereas the in-hospital death rate among persons with advanced AIDS is 29% [12]. Our study showed that the risk of death was significantly lower for patients in rural health zones compared to those in urban areas. There is not enough research-based evidence concerning epidemiological, clinical, social, and environmental factors associated with urban–rural differences in mortality in TB-HIV patients. However, we believe several explanations are plausible. TB treatment services are better organized, diseases are well monitored, and community-based surveillance approaches work better in rural areas than in urban areas. The rural residents tend to have more active lifestyles and those with higher severity of illness due to other comorbidities are likely to move to the city and live with relatives for care. In urban areas, self-medication, resistance to antibiotics is prevalent and alternative healers tend to demotivate patients from getting on ART or/and continuing it.

Our study findings showing higher odds of LTFU in patients younger than 15 years is also noteworthy given that the LTFU in children was recently reported to result in higher risk of mortality [32]. Although data on TB/HIV co-infection in children and adolescents are sparse, adherence to ART and delayed notification of serological status in children in the DRC are formally linked to the consent of their guardians or parents. Many patients under 15 are orphans and are at the mercy of their guardians for care. Guardians’ inability or lack of interest in children’s adherence to care, frequent change of residence, and other factors can lead guardians to inadvertently or intentionally discontinue the treatment in the children. These results corroborate with the programmatic data of the PNLS which indicate that very few children are recruited into the active cohort of the PMTCT cascade from pregnant HIV+ women and exposed children born to these HIV+ women (less than 40% of children expected from the 2020 spectrum). Of these exposed children, very few (<10%) are detected early (at 4–6 weeks after birth); with high attrition upon enrollment in the processing department.

Our study has several limitations. First, the data included in this study were cross-sectional. Only the last viral load date and last viral load result were recorded in the dataset, which did not allow us to determine whether viral load results varied for patients during the study period. Patients receiving ART are eligible for viral load testing within 6 months of the ART initiation date and then every 12 months. However, many challenges prevent patients from conforming to this schedule. Additional viral load testing results could have allowed a better measure of variations among factors such as sustained viral load suppression, adherence to recommended viral load test schedule, adherence to ART, and appropriately prescribed ART [33]. Secondly, we found significant variation in missing values among the clinical and patient factors associated with viral load suppression.

Another limitation was that our dataset did not include socioeconomic status measures such as income, education, and assets, which are important predictors of seeking and sustaining medical treatments, especially in resource-limited countries. For instance, Teixeira de Siqueira-Filha and colleagues concluded that patients with TB/HIV coinfection had a higher economic burden than patients with HIV only and also that death resulting from TB/HIV coinfection can cost almost three times more than a death attributable to AIDS [34]. Thus, extremely poor patients may decide to purchase food instead of medication. Additional organizational characteristics are important to account for because several of those characteristics can hinder TB and HIV epidemic control.

This research was based on secondary data from two provinces, which received funding from CDC, and the project implementation partners shared these data. The data from other provinces were not available for this study, posing some limits on the generalizability of our findings. However, the selected two provinces are diverse, supporting the generalizability of our results to several other parts of the country not covered in this study. For instance, the province of Kinshasa covers a greater proportion of the urban population and features greater cultural diversity compared to Haut-Katanga which is predominantly rural. Finally, the dependent variable of interest, TB/HIV coinfection, could have been underreported or underdiagnosed. To assess sustained viral load suppression, future studies could consider using multiple-year data from more geographic locations and could also consider collecting additional data to control for socioeconomic status variables (e.g., income, education/literacy, or home-ownership) that could confound clinical characteristics and mortality results. Regardless of these limitations, our study has strengths resulting in significant contributions to the body of scientific evidence available to HIV programs in DRC. Our findings coupled with the emerging evidence show that patients with TB/HIV coinfection require special attention and treatment, including TB-HIV integrated care and specific first-line therapies (e.g., dolutegravir) to improve treatment outcomes [35,36]. Our study utilizes a robust sample size to allow stable estimates of the influence of TB/HIV coinfection on outcome variables, after controlling for several sociodemographic and clinical variables included in the multivariable analysis. To our knowledge, such a study of these DRC data has not been conducted in recent years. Finally, our study exemplifies the practice-based research using secondary data in that researchers and program partners collaboratively completed this study from conceptualization to study’s completion.

Our study showed that 3.6% of patients receiving ART have TB/HIV coinfection. TB/HIV coinfection significantly increased the risk of death and LTFU and decreased viral load suppression rates. Our findings suggest that the interventions and programs for PLWH in Kinshasa and Haut-Katanga provinces could benefit from quality improvement and active research via tuberculosis surveillance systems. Understanding and addressing social stigma and clinical challenges faced by patients with TB/HIV coinfection, including the use of counseling and integrated treatment for HIV and TB, could help improve healthcare and outcomes.

## 5. Conclusions

This study of a rather large patient population showed that TB/HIV coinfection is found in 3.6% of patients on antiretroviral therapy in the 241 clinics that shared data for this study. TB/HIV coinfection was found to significantly raise the risk of negative outcomes (death, loss to follow-up, and no viral load suppression). The study suggests that understanding and addressing social stigma and clinical challenges faced by TB/HIV co-infected individuals may be beneficial to this group. The findings also suggest that the PLWH interventions and programs in Kinshasa and Haut-Katanga provinces should boost up their quality improvement efforts, and conduct research via tuberculosis surveillance systems in addition to HIV surveillance systems. Patients with TB/HIV coinfection require special attention and treatment, including TB-HIV integrated care and specific first-line therapies to improve treatment outcomes.

## Figures and Tables

**Table 1:** Descriptive statistics for demographic and clinical characteristics of patients (n = 49,460) in HIV/AIDS clinics in Haut-Katanga and Kinshasa provinces, Democratic Republic of Congo, January 2014–May 2019.

Characteristics	N	Percent
**Patient outcome**		
In care, transferred out, or LTFU	46,609	94.2
Death	2851	5.8
Patient Outcome		
In care or transferred out	38,872	83.4
LTFU	7737	16.6
**Viral load suppression (<1000 copies per mL)**		
Not suppressed	2852	18.8
Suppressed	12,281	81.2
Patient sex		
Female	34,134	69.0
Male	15,326	31.0
**Age at the time of first visit, years**		
<15	4769	10.3
≥15 years	41,454	89.7
TB status of patient		
No TB	43,218	96.4
TB present	1631	3.6
**ART initiation mode**		
New patient	44,060	95.2
Transferred in	2214	4.8
**Duration on ART, months**		
<3.23	11,566	25.0
3.23–14.52	11,540	25.0
14.53–40.37	11,549	25.0
>40.37	11,542	25.0
**Province**		
Haut-Katanga	14,596	29.5
Kinshasa	34,864	70.5
**Rural/urban status of the health zone**		
Rural	2790	5.6
Semi-rural	5780	11.7
Urban	40,890	82.7
**Health zone**		
Binza Ozone	2453	5.0
Kafubu	299	0.6
Kasenga	737	1.5
Kashobwe	73	0.1
Katuba	2688	5.4
Kilwa	498	1.0
Kimbanseke	1788	3.6
Kingabwa	2510	5.1
Kinshasa	2220	4.5
Kipushi	2091	4.2
Kisanga	2822	5.7
Kowe	229	0.5
Limete	3498	7.1
Lingwala	4786	9.7
Masina I	4429	9.0
Matete	1888	3.8
Mont Ngafula 1	1854	3.7
Mumbunda	2390	4.8
Ndjili	4693	9.5
Ngaba	2910	5.9
Nsele	1835	3.7
Pweto	1183	2.4
Tshamilemba	1586	3.2

Abbreviations: TB, Tuberculosis; ART, antiretroviral therapy; LTFU, lost to follow-up.

**Table 2:** Bivariate association between demographic/clinical characteristics and TB coinfection among patients (n = 49,460) in HIV/AIDS clinics of Haut-Katanga and Kinshasa provinces, Democratic Republic of Congo, January 2014–May 2019.

Clinical and Demographic Characteristics	TB Status		
	No TB	TB Present	*p*-Value
	n (%)	n (%)	
**Patient Sex**			<0.001
Female	29,879	1008	
	(96.7)	(3.3)	
Male	13,339	623	
	(95.5)	(4.5)	
**Age at the time of first visit**			0.450
Younger than 15 years	4415	157	
	(96.6)	(3.4)	
15 years or older	38,781	1471	
	(96.3)	(3.7)	
**ART Initiation Mode**			<0.001
New Patient	41,106	1594	
	(96.3)	(3.7)	
Transferred In	2101	35	
	(98.4)	(1.6)	
**Duration on ART (months)**			<0.001
Less than 3.23 months	10,663	661	
	(94.2)	(5.8)	
3.23 to 14.52 months	10,735	459	
	(95.9)	(4.1)	
14.53 to 40.37 months	10,813	295	
	(97.3)	(2.7)	
More than 40.37 months	10,976	213	
	(98.1)	(1.9)	
**Province**			<0.001
Haut-Katanga	12,679	358	
	(97.3)	(2.7)	
Kinshasa	30,539	1273	
	(96.0)	(4.0)	
**Rurality/Urbanicity of the Health Zone**			<0.001
Rural	2643	33	
	(98.8)	(1.2)	
Semi-rural	5381	170	
	(96.9)	(3.1)	
Urban	35,194	1428	
	(96.1)	(3.9)	
**Health Zone**			<0.001
Binza Ozone	1997	107	
	(94.9)	(5.1)	
Kafubu	296	3	
	(99.0)	(1.0)	
Kasenga	615	13	
	(97.9)	(2.1)	
Kashobwe	73	0	
	(100.0)	(0.0)	
Katuba	1863	95	
	(95.1)	(4.9)	
Kilwa	496	2	
	(99.6)	(0.4)	
Kimbanseke	1739	18	
	(99.0)	(1.0)	
Kingabwa	2448	11	
	(99.6)	(0.4)	
Kinshasa	1952	60	
	(97.0)	(3.0)	
Kipushi	1923	48	
	(97.6)	(2.4)	
Kisanga	2531	63	
	(97.6)	(2.4)	
Kowe	229	0	
	(100.0)	(0.0)	
Limete	3183	184	
	(94.5)	(5.5)	
Lingwala	3798	229	
	(94.3)	(5.7)	
Masina I	3896	84	
	(97.9)	(2.1)	
Matete	1704	93	
	(94.8)	(5.2)	
Mont Ngafula 1	1732	68	
	(96.2)	(3.8)	
Mumbunda	1938	92	
	(95.5)	(4.5)	
Ndjili	3722	238	
	(94.0)	(6.0)	
Ngaba	2642	127	
	(95.4)	(4.6)	
Nsele	1726	54	
	(97.0)	(3.0)	
Pweto	1163	15	
	(98.7)	(1.3)	
Tshamilemba	1552	27	
	(98.3)	(1.7)	

Abbreviations: TB, Tuberculosis; ART, antiretroviral therapy.

**Table 3:** Logistic regression of patient outcomes among patients (n = 49,460) in HIV/AIDS clinics of Haut-Katanga and Kinshasa provinces, Democratic Republic of Congo, January 2014–May 2019.

Characteristics	Patient Outcome—Death (vs. In Care, Transferred Out, or LTFU)			Patient Outcome—LTFU (vs. In Care or Transferred Out)			Suppressed Viral Load (vs. Not Suppressed)		
	AOR	95% CI for		AOR	95% CI for		AOR	95% CI for	
		Lower	Upper		Lower	Upper		Lower	Upper
TB status									
TB present	**2.26**	1.94	2.64	**2.06**	1.82	2.34	**0.58**	0.46	0.74
No TB	**--**	--	--	**--**	--	--	**--**	--	--
Patient sex									
Female	**0.36**	0.34	0.39	**0.72**	0.68	0.76	**1.82**	1.68	1.96
Male	**--**	--	--	**--**	--	--	**--**	--	--
Age at the time of first visit, years									
<15	1.1	0.97	1.25	**1.25**	1.14	1.37	**0.44**	0.39	0.5
≥15	**--**	--	--	**--**	--	--	**--**	--	--
ART initiation mode									
New patient	**0.08**	0.07	0.09	**0.15**	0.14	0.16	**2.16**	1.99	2.34
Transferred in	**--**	--	--	**--**	--	--	**--**	--	--
Duration on ART, months									
<3.23	**2.32**	2.08	2.59	**2.83**	2.61	3.08	0.82	0.5	1.34
3.23–14.52	**0.81**	0.71	0.93	**1.35**	1.24	1.48	**1.35**	1.18	1.55
14.53–40.37	**0.59**	0.52	0.67	0.98	0.9	1.08	**1.28**	1.16	1.4
>40.37	**--**	--	--	**--**	--	--	**--**	--	--
Province									
Haut-Katanga	**1.14**	1.04	1.25	**0.5**	0.46	0.54	**2.04**	1.82	2.28
Kinshasa	**--**	--	--	**--**	--	--	**--**	--	--
Rural/urban status of the health zone									
Rural	**0.24**	0.17	0.33	**0.04**	0.02	0.08	**1.5**	1.15	1.96
Semi-rural	**1.2**	1.07	1.35	**0.51**	0.46	0.57	1.07	0.94	1.21
Urban	**--**	--	--	**--**	--	--	**--**	--	--

Abbreviations: LTFU, lost to follow-up; AOR, adjusted odds ratio; CI, confidence intervals; TB, tuberculosis; ART, antiretroviral therapy. Note: Bold indicates a statistically significant difference at *p* < 0.05.

## Data Availability

The program implementing partners required that data be destroyed after publication. The authors do have data till the publication of the article. The authors can facilitate data access if requested with proper permission from the DRC Ministry of Health.
